# Predicting hypertension control using machine learning

**DOI:** 10.1371/journal.pone.0299932

**Published:** 2024-03-20

**Authors:** Thomas Mroz, Michael Griffin, Richard Cartabuke, Luke Laffin, Giavanna Russo-Alvarez, George Thomas, Nicholas Smedira, Thad Meese, Michael Shost, Ghaith Habboub

**Affiliations:** 1 Orthopaedics and Rheumatology Institute, Cleveland Clinic, Cleveland, OH, United States of America; 2 Center for Spine Health, Cleveland Clinic, Cleveland, OH, United States of America; 3 Insight Enterprises Inc., Chandler, AZ, United States of America; 4 Department of Internal Medicine, Cleveland Clinic, Cleveland, OH, United States of America; 5 Department of Cardiovascular Medicine, Center for Blood Pressure Disorders, Cleveland Clinic, Cleveland, OH, United States of America; 6 Department of Hospital Outpatient Pharmacy, Cleveland Clinic, Cleveland, OH, United States of America; 7 Department of Kidney Medicine, Cleveland Clinic, Cleveland, OH, United States of America; 8 Department of Thoracic and Cardiovascular Surgery, Cleveland Clinic, Cleveland, OH, United States of America; 9 Department of Innovations Technology Development, Cleveland Clinic, Cleveland, OH, United States of America; 10 Case Western Reserve University School of Medicine, Cleveland, OH, United States of America; Qatar University, QATAR

## Abstract

Hypertension is a widely prevalent disease and uncontrolled hypertension predisposes affected individuals to severe adverse effects. Though the importance of controlling hypertension is clear, the multitude of therapeutic regimens and patient factors that affect the success of blood pressure control makes it difficult to predict the likelihood to predict whether a patient’s blood pressure will be controlled. This project endeavors to investigate whether machine learning can accurately predict the control of a patient’s hypertension within 12 months of a clinical encounter. To build the machine learning model, a retrospective review of the electronic medical records of 350,008 patients 18 years of age and older between January 1, 2015 and June 1, 2022 was performed to form model training and testing cohorts. The data included in the model included medication combinations, patient laboratory values, vital sign measurements, comorbidities, healthcare encounters, and demographic information. The mean age of the patient population was 65.6 years with 161,283 (46.1%) men and 275,001 (78.6%) white. A sliding time window of data was used to both prohibit data leakage from training sets to test sets and to maximize model performance. This sliding window resulted in using the study data to create 287 predictive models each using 2 years of training data and one week of testing data for a total study duration of five and a half years. Model performance was combined across all models. The primary outcome, prediction of blood pressure control within 12 months demonstrated an area under the curve of 0.76 (95% confidence interval; 0.75–0.76), sensitivity of 61.52% (61.0–62.03%), specificity of 75.69% (75.25–76.13%), positive predictive value of 67.75% (67.51–67.99%), and negative predictive value of 70.49% (70.32–70.66%). An AUC of 0.756 is considered to be moderately good for machine learning models. While the accuracy of this model is promising, it is impossible to state with certainty the clinical relevancy of any clinical support ML model without deploying it in a clinical setting and studying its impact on health outcomes. By also incorporating uncertainty analysis for every prediction, the authors believe that this approach offers the best-known solution to predicting hypertension control and that machine learning may be able to improve the accuracy of hypertension control predictions using patient information already available in the electronic health record. This method can serve as a foundation with further research to strengthen the model accuracy and to help determine clinical relevance.

## Introduction

The prevalence of hypertension, defined as blood pressure (BP, mmHg) ≥130 systolic or ≥ 80 diastolic, is estimated to be 116 million Americans and 1.3 billion people globally [1]. It remains the leading national and global cause of cardiovascular disease and premature death. In the U.S., hypertension is associated with 400,000 deaths per year, or about 1000 deaths per day, and worldwide the disease is attributed to 7.6 million deaths each year. Hypertensive patients are at approximately 2–4 times higher risk for coronary heart disease, stroke, peripheral vascular disease, and congestive heart failure [2]. The associated financial tolls are staggering. By 2035, it is estimated that cardiovascular disease will cost the U.S. annually in direct and indirect expense $1.1 trillion dollars, and hypertension will account for over $220 billion [3].

Less than one-third of the U.S. population with hypertension are controlled [4]. There are many reasons for poor BP control, including patient education, adherence to pharmacotherapy, and medication and dose selection. Computing capacity has increased exponentially over the past several decades, and types of artificial intelligence, such as machine learning (ML) and deep learning have been applied to cardiovascular medicine. Both traditional regression-based methods [5–10] and ML [11–16] have been successfully used to predict incident hypertension, but no studies have applied ML to predict future control in hypertensive patients. The ability to predict control at any point in a patient’s care could shorten the time to control by triggering alternative approaches to care including medication and/or dose changes. The less time patients are hypertensive has been associated with lower cardiovascular and cerebral risk [17–22]. Therefore, quicker achievement of BP control could potentially diminish morbidity and mortality if an accurate prediction of control is coupled to a change in therapy.

This project used ML to predict BP control at 12 months in hypertensive patients. The hypothesis was that ML would render an accurate prediction of control at various future timepoints using large patient data repositories within our health system.

## Methods

### Study design

This is a retrospective study designed to determine if ML can be used to predict hypertension control 12 months from a clinical encounter.

### Data source

All included patients were outpatients and diagnosed with hypertension ICD-10 codes I10-I15 between January 1, 2015, and June 1, 2021, and were 18 years of age and older. Data for this project was accessed on 10/18/2022 following approval (protocol #22–1139) from the Cleveland Clinic Institutional Review Board and patient data was reviewed in aggregate without identification of individual patient data. All outpatient electronic health record (EHR) data collected up to and including the current clinical visit were organized into a single row of data for each patient and for each of their clinical visits. However, this does not mean that all data was combined into a single value. Some data was aggregated, for example, maximum systolic pressure over the previous 12 months. Other data was kept as is, for example, current systolic pressure. The EHR used here did not include an explicit designation that a patient’s hypertension was controlled or not. Per American College of Cardiology (ACC) and American Heart Association (AHA), who define hypertension as “blood pressure consistently at or above 130/80 mmHg,” in this study, patients with systolic BP above 130 mmHg or diastolic BP above 80 mmHg were defined as not having their blood pressure controlled [1]. Patients were required to have their first and last encounters separated by at least 12 months such that ‘control’ could be predicted 12 months from the date of the earlier encounter. As a result, the last year of patient data from June 1, 2021, to June 1, 2022 was only used to determine control. Using this cohort, a large EHR data model compatible machine learning algorithms was assembled. The model was structured around clinical encounters where hypertensive patients had their blood pressure measured, and at which they were prescribed one or more anti-hypertensive medications. The ML algorithm incorporated all the structured outpatient EHR data that a clinician would have had access to when they were determining BP treatment options.

### Variables and data analysis

The data considered for this research was all structured outpatient data up to and including data collected in the clinical visit at which a prediction was going to be made. A total of 793 variables were included in the model from the EHR data including vital signs, medications, comorbidities, laboratory results, left ventricular ejection fraction, body mass index (BMI), and outpatient clinical encounters (S1 Table). Vitals, medications, and outpatient clinical encounters were further processed to incorporate temporal changes of each into the data model to provide a dynamic representation of each patient’s data and enable the algorithm to find patterns, trends, and fluctuations in health indicators, leading to more accurate predictions. Importantly, the calculated temporal features expose how each patient’s weight has changed, the minimum and maximum BP measurements, and the number of medication changes that a patient had in the last 3, 6 and 12 months. The algorithm then determines if such patterns and trends are predictive of hypertension control. Similar calculations were done for several features of clinical encounters. These calculations are also called feature engineering, which is typically done to improve predictive accuracy. Categorical variables were one-hot encoded, where a field with multiple options, e.g. patient gender, race, or ethnicity were converted to a form where each possible response was represented by a separate data column with a value of ‘1’ assigned to the corresponding features for each patient. One-hot encoding and temporal feature engineering added nearly six hundred columns of data to the data model. While there are other factors important for consideration to hypertension such as patient education, adherence tendencies, presence of drug interactions, exposure to adverse side effects, financial status, social history, or lifestyle, this information was not available and thus was not included in the model.

The predictive model developed here used the XGBoost algorithm. XGBoost is one of the most popular machine learning algorithms. It is an open source, platform-independent, efficient, and accurate boosted tree ensemble method. XGBoost has been used successfully for research and production in every industry including healthcare. To support missing data the XGBoost algorithm uses a technique called "sparsity aware split finding". XGBoost treats missing values as distinct entities and learns optimal decision-making procedures in their presence. This methodology, coupled with regularization techniques enables XGBoost to efficiently handle datasets with missing values, which allows for the inclusion of patients with very little data/history as well as those patients with long histories. Another useful feature of the algorithm is the availability of several parameters that can be tuned to avoid overfitting predictive models. Overfitting models can lead to misleadingly good performance in the test environment followed by poor performance in a production environment. Considerable care was taken in the current research to avoid overfitting the machine learning models.

A comprehensive grid search technique was performed to tune the XGBoost hyperparameters. The sliding window technique described above, where the model was retrained and tested on 287 different windows of data with the same set of hyperparameters, provided a method to assess how well the model would generalize to new, unseen data. This approach is very similar to cross validation techniques, i.e., each window can be considered a "fold" as in k-fold cross-validation. The mean AUC for these models is 0.756 with a standard deviation of 0.026 indicating a robust set of hyperparameters that are not prone to overfitting the data. These aggregated model performance metrics are augmented by the uncertainty analysis described here which gives insight into the reliability of individual predictions.

Results were described as area under the curve (AUC), sensitivity, specificity, positive predictive value (PPV), and negative predictive value (NPV). The AUC is a widely used performance metric in machine learning, particularly for binary classification tasks. It provides an evaluation of the model’s ability to discriminate between two classes, such as identifying patients with and without hypertension control, and represents the overall performance of the model across all possible thresholds. The AUC ranges from 0 to 1, where a higher value indicates a higher number of correctly identified blood pressure control. The AUC was derived from a receiver operating characteristic (ROC) curve, which plotted the true positive rate (sensitivity) against the false positive rate (1—specificity) at various classification thresholds. In other words, sensitivity (or, recall) displayed the ability of the model to label positive control outcomes as positive, and specificity showed the ability of the model to label negative control outcomes as negative, where both were plotted in the ROC curve. High sensitivity and specificity scores indicate that the model predicts BP control at 12 months in hypertensive patients well. The PPV is the ratio of blood pressure correctly identified as controlled by the ML model to all controlled blood pressure actually present in the data. Similarly, the NPV is the ratio of blood pressure identified as not controlled by the ML model to all blood pressure non-control actually in the data.

### Machine learning algorithm and technology

All data engineering and data science were performed on a standard virtual machine in Google Cloud Platform. Specialized computing hardware such as graphics processor units (GPUs) was not required or used. Python and SQL were the only programming languages used. Computer code was written in Jupyter notebooks and output was written to image files and comma separated values files.

## Modeling challenges

### Data drift

**Fig 1:**
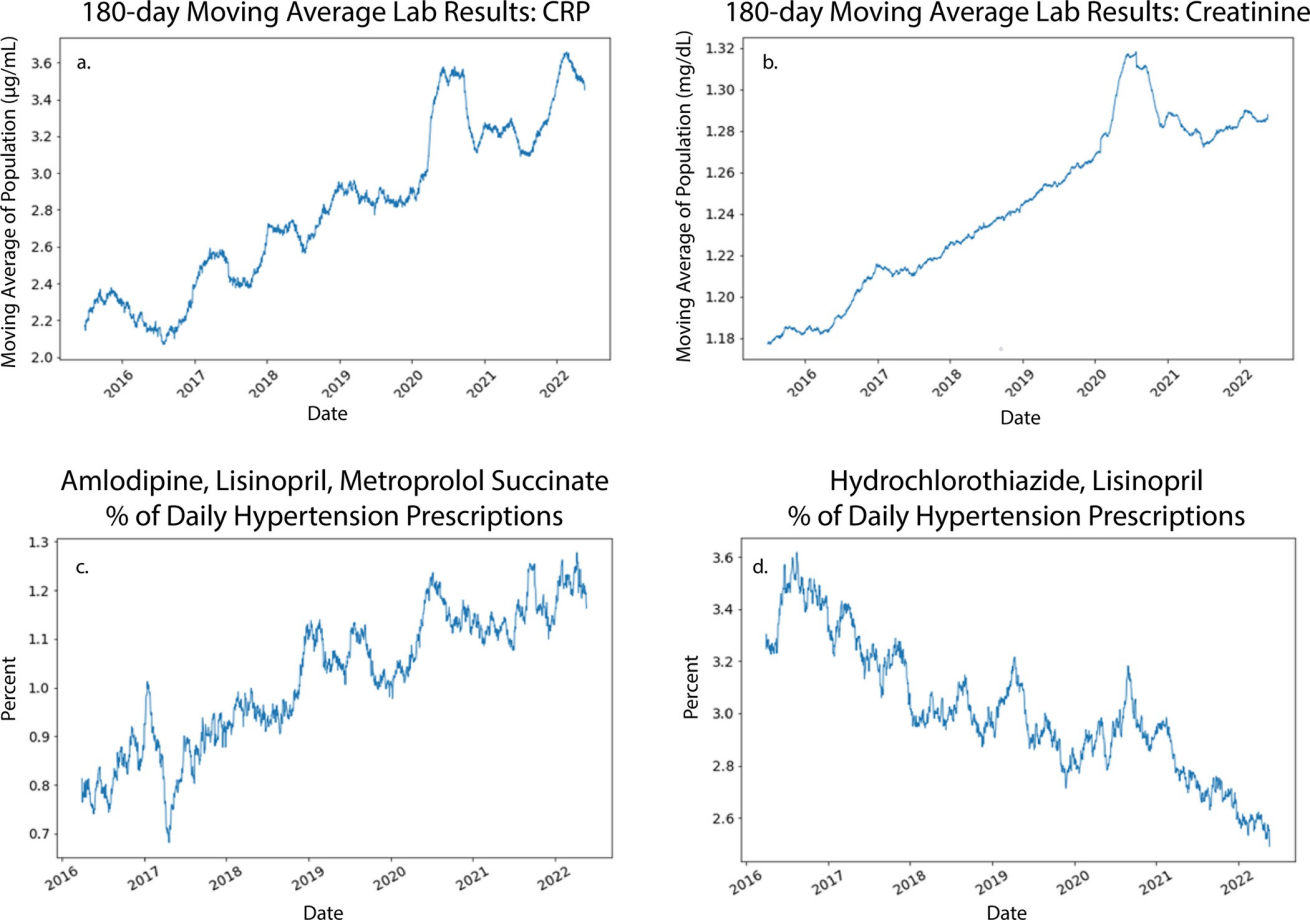
Examples of data drift due to changes in patient population health and changes in the treatment of patients. (a) & (b) show the 180-day moving average of all c-reactive protein and creatinine lab results for the hypertensive patients considered here. The average c-reactive protein lab result has increased by 60% over the last seven years. The average creatinine result has increased by more than 6% over the same period. Nearly all quantitative lab results exhibit similar drift, indicating a significant decline in the health of the hypertension patient population. (c) & (d) show two examples of treatment drift. The 30-day moving average of the percentage of all hypertension medication orders that were amlodipine, lisinopril & metoprolol succinate or hydrochlorothiazide & lisinopril during the period January 2015 to June 2022. The combination of amlodipine, lisinopril & metoprolol succinate has been slowly increasing in popularity relative to all other combinations, while the combination of hydrochlorothiazide & lisinopril has been slowly decreasing.

‘Data drift’ is defined as a temporal change in the mean of the EHR data used to train and test the ML models. ML models learn patterns in data, and if data drift changes the patterns in the data, model performance may degrade. Two types of data drift were identified in the EHR which we designate as 1) patient data drift and 2) treatment data drift. Two examples of patient data drift and two examples of treatment drift are shown in Fig 1. The moving average of all c-reactive protein and creatinine laboratory results (Fig 1(A) & 1(B), respectively) rose significantly during the study period. The frequency of prescribing the combination of amlodipine, lisinopril and metoprolol succinate increased, while the frequency of prescribing the combination of hydrochlorothiazide and lisinopril decreased as shown in Fig 1(C) & 1(D), respectively. Other examples of both patient data and treatment data drift were identified but are not shown. Both types of data drift significantly degrade ML model performance. In practice, the impact of data drift on model performance is mitigated by periodically retraining ML models on the newest data.

To accommodate for drift, the models were retrained every week. This technique allows the inclusion of new data, which is drifting, every week. This required only a few seconds on a standard 96 CPU virtual machine in Google Cloud Platform that was used for this project. This is a very inexpensive, automated, and sustainable method to accomplish the retraining.

#### Blood pressure variability

**Fig 2:**
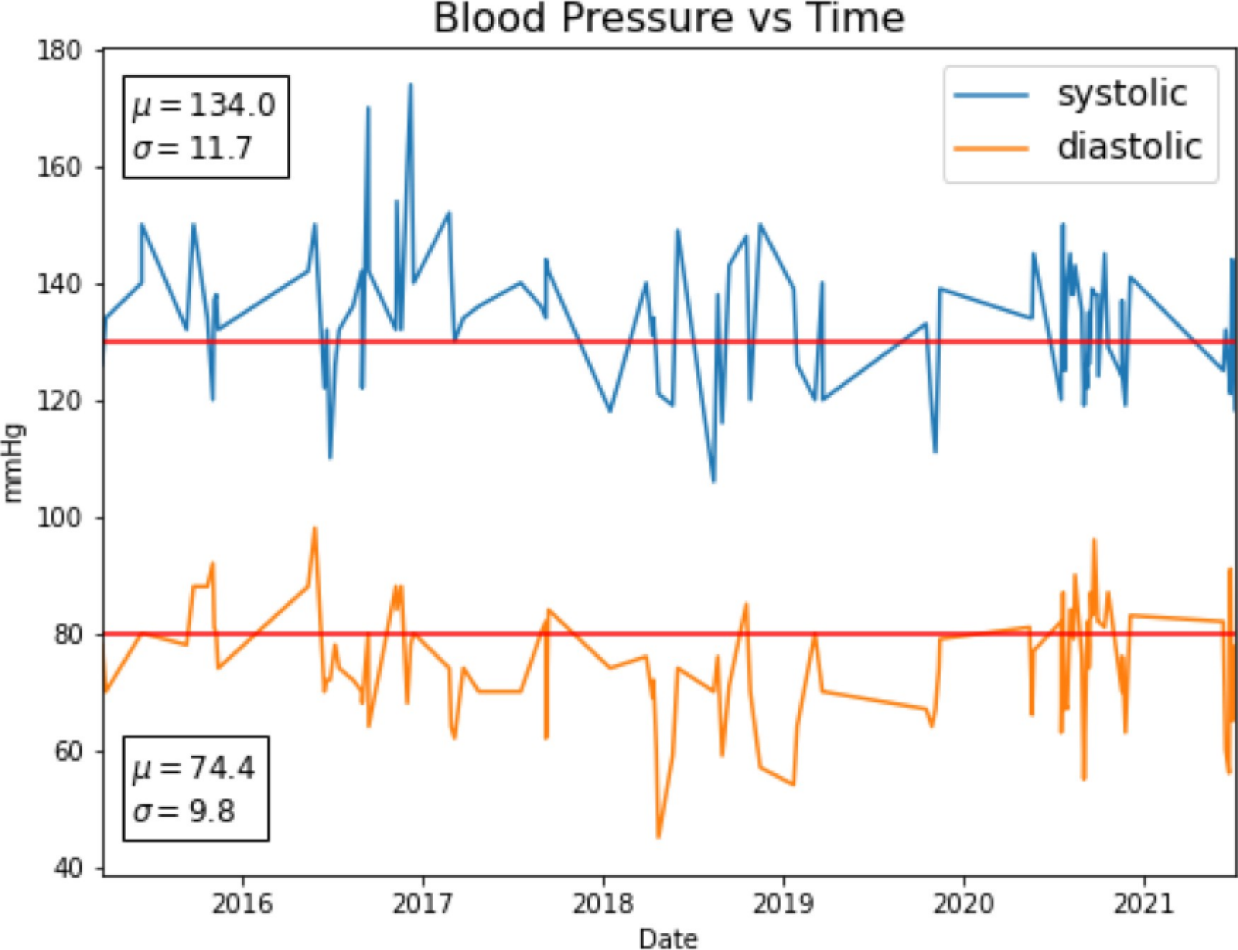
The blood pressure history of a single patient plotted as a time series. The horizontal red lines represent the guidelines that define hypertension. If a patient’s systolic pressure is above 130 mmHg or diastolic pressure is above 80 mmHg, then the patient has uncontrolled hypertension. The mean and standard deviation of each measure are also shown.

Individual variability in office BP measurements presented another significant challenge in predicting control of hypertension. Fig 2 shows the blood pressure history of a typical patient. Fluctuations may obscure patterns such as increasing or decreasing blood pressure. For example, using our definition of control, this patient might be controlled and then not controlled within very short time windows on multiple occasions. It is unlikely that any ML model using any algorithm will correctly predict all such fluctuations.

#### Model uncertainty

**Fig 3:**
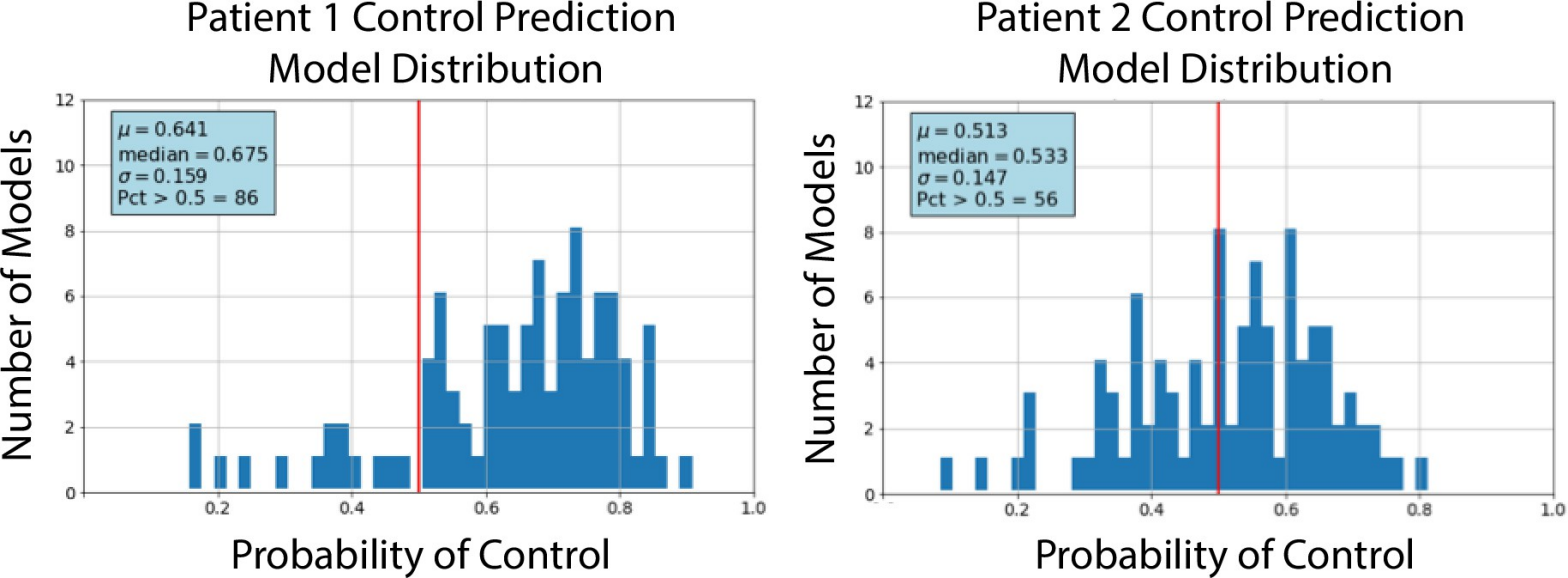
The distribution of 100 probabilities that two separate patients will have their hypertension controlled within 12 months. The probabilities were obtained from 100 ML models each trained with different subsets of EHR data. The mean, median, standard deviation, and the percentage of probabilities above 0.5 are shown in the blue boxes. The vertical red lines indicate the prediction threshold–probabilities above 0.5 result in a prediction that the patient’s hypertension will be controlled. The mean probabilities indicate that both patients would be predicted to have their hypertension controlled, but the prediction that Patient 1 will have their hypertension controlled appears to be more certain than the prediction that Patient 2 will have their hypertension controlled.

ML models are typically evaluated on their performance against an entire population, e.g., AUC, specificity, and sensitivity. The uncertainty associated with individual predictions is not reflected in these aggregated statistics. Small changes in training data subsets that result in very small changes to aggregated model statistics can result often in substantial changes to individual predictions. To help understand the uncertainty of individual predictions, 100 training data sets were prepared by randomly sampling the complete data set. These training data sets were used to train 100 ML models, which were then used to predict hypertension control 100 times for each patient. As an example, Fig 3 shows the distribution of prediction probabilities for two patients using 100 models. Also listed in the figure are the mean, median, standard deviation, and percentage of probabilities above 0.5. One interpretation of this figure is that while both patients are predicted to have their hypertension controlled, the prediction for Patient 2 has a higher uncertainty.

## Results

The training and validation cohorts consisted of a total of 350,008 hypertensive patients and 10,564,174 outpatient clinical encounters during the study period. Of the 10,564,174 encounters, 1,543,461 included patient BP measurements and medication prescriptions to treat hypertension. The remaining 9,020,713 encounters were not explicitly related to treating hypertension. Information about the non-hypertension related encounters, such as counts of encounter types (e.g., primary care provider visits) was incorporated into the ML data model as an additional column, (e.g., “count of PCP encounters”). This technique allowed us to incorporate a measure of resource utilization in the data model, which may be predictive of blood pressure control. The resulting ML data set consisted of 1,543,461 rows and over 800 columns. 43.5% of hypertensive patients had their hypertension controlled within 12 months of a previous encounter.

**Fig 4:**
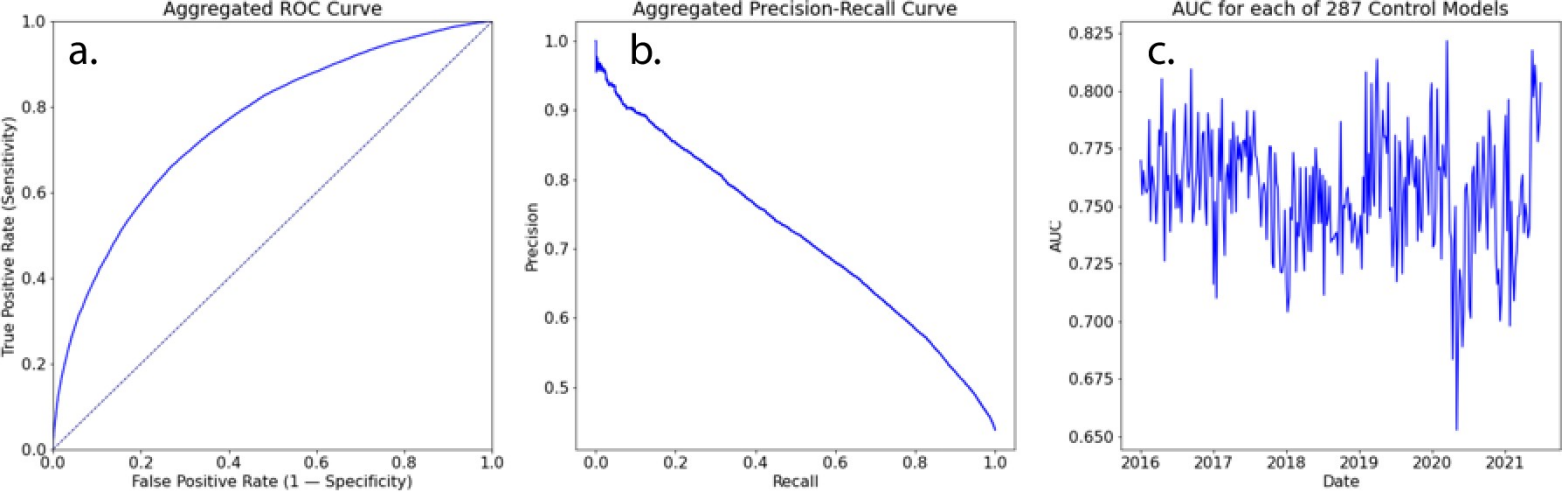
(a) ROC and (b) PR curves and (c) the Area Under the Curve (AUC) for the 287 generated ML models. Mean AUC is 0.756 with a standard deviation of 0.026. The model was retrained every week and the ROC and PR curves represent the aggregated ROC & PR for all models. Fig 4(c) shows the AUC for each of the models.

To maximize the performance of the ML models and address data drift, the complete data set was split many times using a sliding window of two years of data to train each model. Each test data set was constructed by taking a fixed time window of data following the last date used in each training data set. For example, one model used data from January 2015 through December 2016 for training and data from 2017 for testing. The window of time for testing was varied from one day to one year to find the optimal retraining cadence. We found that retraining once per week was optimal, which resulted in training and testing a total of 287 ML models. Fig 4(A) and 4(B) shows the ROC and the precision-recall curves for the aggregation of all models.

The AUC for this aggregation is 0.756. Training so many ML models provides the ability to evaluate whether the ML models are overfitted and is effectively a cross validation technique with 287 “folds”. AUC vs time for each of 287 ML models is shown in Fig 4(C). While there are fluctuations, e.g., at the onset of the COVID-19 pandemic, model performance was very consistent across more than five years of simulated retraining, indicating that the models are unlikely to be overfitted.

Table 1 lists the feature importance as measured by the gain for several categories of features for one of the 287 control models.

**Table 1:** Feature importance by category for a representative control model. The magnitude of the importance changes from model to model. For the XGBoost algorithm, gain is a measure of the improvement in accuracy that a feature delivers when present on a decision tree.

**Medication Combination**	**Gain**
Chlorthalidone, metoprolol tartrate, spironolactone	18.0
Furosemide, lisinopril, nadolol, spironolactone	13.4
Carvedilol, furosemide, metolazone, spironolactone	12.4
Carvedilol, chlorthalidone, lisinopril	10.9
Carvedilol, chlorthalidone, furosemide, lisinopril	10.4
Carvedilol, furosemide, spironolactone	10.4
Hydrochlorothiazide, metoprolol succinate, triamterene	10.2
Hydrochlorothiazide, metoprolol succinate, nifedipine, spironolactone, valsartan	9.7
Carvedilol, furosemide, lisinopril, spironolactone, triamterene	9.5
Lisinopril, timolol maleate, triamterene	9.5
**Lab**	**Gain**
Calcium ionized, whole blood	14.0
Carboxyhemoglobin, arterial	11.1
Methemoglobin, venous	10.9
O2 liters per minute	10.1
FiO2	10.0
Respiratory rate	9.9
Absolute eosinophil	9.7
Oxyhemoglobin, venous	9.6
Carboxyhemoglobin, venous	9.6
Bicarbonate, arterial	9.5
Urobilinogen urinalysis (POCT)	9.4
**Vitals**	**Gain**
Systolic min	24.9
Systolic max	19.7
Diastolic min	13.1
Diastolic max	11.8
Systolic spread	9.2
Diastolic spread	9.0
Diastolic standard deviation	9.0
Systolic over diastolic	8.9
Systolic standard deviation	8.8
Systolic diastolic spread	8.5
**Comorbidity**	**Gain**
Adverse cardiovascular event	9.9
Congestive heart failure	9.7
Limited life expectancy	9.4
Number of comorbidities	8.9
Mental health	8.3
COPD	8.2
Diabetes	8.0
Stroke	7.8
Malignant cancer	7.8
Myocardial infarction	7.6
ESRD	4.2
**Encounter**	**Gain**
History 90-day mean count	14.7
Telephone 30-day mean count	11.4
Office visit 90-day mean count	11.2
Appointment 180-day mean count	10.2
Anticoagulation visit 180-day mean count	10.2
Telephone count	10.0
Radiology 180-day mean over 90-day mean count	9.7
Appointment 30-day mean count	9.6
Billing encounter 180-day mean count	9.6
Refill CP count	9.5
Office visit count	9.4
**Language**	**Gain**
Spanish	8.6
Arabic	8.5
Russian	8.1
English	8.0
Other	7.2
Ukrainian	6.6
Unknown	6.6
Cantonese	6.3
Italian	5.6
Hindi	5.6
Sign Language	5.4
**Census**	**Gain**
Family median income	9.4
Nonfamily households total $200k+	9.2
Households mean income	9.2
Households total $25k to $35k	9.2
Married family total	9.2
Households total $50k to 75k	9.1
Households total $200k+	9.1
Married family total $150k to $200k	9.1
Nonfamily households total $100k to $150k	9.1
Married family total $15k to $25k	9.1
Married family median income	9.0

## Discussion

The purpose of this study was to determine if ML could predict future BP control from a clinical encounter in hypertensive patients. It is well-known that controlling hypertension [23–28] and decreasing the time to control [17–22] is associated with diminished cardiovascular risk. It is logical to postulate that early prediction of BP control may improve time to control if the prediction prompts a change to more effective therapy. Machine learning [29–32] and deep learning [33–39] have emerged to show promise in the management of various cardiovascular diseases, and ML has been used to predict incident hypertension in a variety of scenarios [11–16]. However, there are no published reports of using ML to predict control in patients currently on hypertensive pharmacotherapy; therefore, this study that includes 350,008 patients and 10,564,174 clinical encounters is the first to do so.

Reducing time to BP control has been shown to reduce morbidity and mortality [17–22]. The ALLHAT (Antihypertensive and Lipid-Lowering Treatment to Prevent Heart Attack Trial) study demonstrated that control in less than 6 months was associated with reduction in stroke, and prolonged hypertension was associated with higher incidence of heart failure and coronary artery disease [21]. The SCOPE (Study On Cognition and Prognosis in the Elderly) trial reported a lower incidence of fatal- and non-fatal stroke in patients who achieved control in less than 3 months [19]. In a post-hoc analysis, the Valsartan Antihypertensive Long-term Use of Evaluation (VALUE) trial reported a significant reduction of combined cardiac events, death, and stroke in patients achieving a systolic BP below 140 mm Hg within 6 months irrespective of treatment arm [22].

Our study is an early step toward using advanced computing to predict control. Through changes in therapy, this could improve the overall time to control. A key finding is that control prediction within 12 months from a clinical encounter has a reasonably high accuracy (0.756 AUC). Although the latter value usually indicates mediocre ML model performance, we believe that this AUC is promising considering the fluctuations in typical office BP measurements and data drift as described previously. As with any ML model with clinical applications, the only way to effectively evaluate its performance in terms of clinical relevancy is to deploy this clinical support tool in practice and study its impact on hypertension control. Current control was determined to be most predictive of future control when compared to over 800 included patient features. Table 1 shows that maximum systolic blood pressure values within the last 12 months were found to have nearly double the predictive importance when compared to maximum diastolic values. Similarly, the combination of chlorthalidone, metoprolol tartrate and spironolactone was nearly twice as predictive of control as the combination of carvedilol, furosemide, lisinopril, and spironolactone. This is not to suggest that the former is more effective than the latter; it simply quantifies the contribution to predictive accuracy when patients are prescribed those medications compared to when they are not.

An advantage of ML with large data repositories, and one deployed in this study, is trend analysis. A binary application of features (e.g., diabetes yes / no) to the control model is an oversimplification that would limit predictive accuracy. Moreover, it would not represent the dynamic interplay and influence that various features have with each other nor their absolute values and rate of change over time. Trends of various features and their influence on predictive accuracy were considered intentionally throughout this study. Among the many trends studied in this report, we found that rising, declining or stable trends of systolic pressure were the feature trends that most predict control, or lack thereof.

Gain was measured intentionally in this study and represents the boost to accuracy of a decision tree when that feature is present in the tree. Gain does not imply that a feature increases or decreases the probability of control. Feature importance can be used improve model interpretations and to suggest patient interventions that may improve outcomes. For example, several types of patient encounters are predictive of control such as the number of recent telephone calls between the hospital and patient. Further investigation may indicate that reaching out to patients for follow-up care may increase the number of patients who control their blood pressure. ML models, like this one, include hundreds and sometimes thousands of features and further investigation is required to define their importance to outcome. This will improve outcome interpretation relative to feature(s) and inform provider decisions to enhance care.

This main contribution of this study is the novel and successful application of ML to predict control from any encounter of hypertensive patients. A strength is the large number of included patients and clinical encounters, and the inclusion of all treating specialties (e.g., Cardiology, Internal Medicine, Family Medicine) which we think provides a “real-world” representation. However, this study has several notable limitations. One, the retrospective review of prospectively compiled institutional data is a fundamental study design limitation. However, given the number of included patients and encounters, a prospective study of this size would not be feasible. Two, this model will require prospective validation of the control predictions and external validation over various geographic hypertensive cohorts. A key to such validation will be to refine and confirm feature importance. Three, the lack of cause and effect clarity of feature importance is a current weakness of ML and this study because of the inability to completely define how certain features influence the prediction. While techniques such as Shapley values can help further define features [40], more research is necessary to understand mechanistic associations of features to outcome. An example is the varied feature importance across medications. This could indicate a phenotypic variation in response to anti-hypertensive medication as reflected by Sundstrom et al.’s recent publication [41]. As another example of feature importance, consider a hypertensive patient with 12-months rising creatinine, stable hemoglobin A1c, increasing BMI, downward trending ejection fraction, and prescribed both amlodipine and hydrochlorothiazide. In such a patient, the control model may indicate a 78% chance of control in nine months. Though, it will not inform why or how these features, their absolute values and rate of change, and their potential interplay with other features are causing or influencing blood pressure at specific timepoints. However, such modeling does permit predictions about control when such a patient is compared to thousands of other hypertensive patients. A fourth limitation is the lack of imaging analysis, such electrocardiogram and echocardiogram. Deep learning was not employed in this study but may offer opportunities to further strengthen hypertension control models. Despite these limitations, we believe this study can serve as a foundation to further develop capacity and define relevance of computing for the treatment of hypertensive patients.

## Conclusion

This is the first study to use ML to predict blood pressure control and models demonstrated reasonably high accuracy in predicting control within 12 months of a patient encounter. The study highlights the potential of ML to decrease the time to control once the models are validated and improved. Further study will be performed to clinically validate the ML models and define applicability.

## Supporting information

S1 Table (DOCX)
