# Supplementary material for: Predicting hypertension control using machine learning
Source: PLoS One. 2024 Mar 20;19(3):e0299932. doi: 10.1371/journal.pone.0299932 (PMC10954144; doi:10.1371/journal.pone.0299932)
Supplement: S1 Table — (DOCX) [file pone.0299932.s001.docx]

**Supplementary Table 1:**

| Machine Learning Variables |
| --- |
| Medication_Order_Dose_Quantity |
| med_days |
| htn_scrip_count |
| htn_scrip_rate |
| htn_med_changes |
| htn_dose_changes |
| htn_dose_over_min |
| htn_dose_over_max |
| htn_dose_over_mean |
| all_scrip_count |
| all_scrip_rate |
| all_med_changes |
| all_dose_changes |
| all_dose_over_min |
| all_dose_over_max |
| all_dose_over_mean |
| htn_med_changes_trend_3 |
| htn_dose_over_mean_trend_3 |
| htn_scrip_rate_trend_3 |
| all_scrip_rate_trend_3 |
| all_med_changes_trend_3 |
| htn_med_changes_trend_6 |
| htn_dose_over_mean_trend_6 |
| htn_scrip_rate_trend_6 |
| all_scrip_rate_trend_6 |
| all_med_changes_trend_6 |
| htn_med_changes_trend_9 |
| htn_dose_over_mean_trend_9 |
| htn_scrip_rate_trend_9 |
| all_scrip_rate_trend_9 |
| all_med_changes_trend_9 |
| TEMP_F |
| PULSE |
| WEIGHT |
| BSA |
| BMI |
| RESP |
| systolic |
| diastolic |
| HEIGHT_inch |
| controlled |
| v_sys_trend |
| v_dia_trend |
| v_bmi_trend |
| v_bsa_trend |
| v_wt_trend |
| v_days |
| v_sys_min |
| v_dia_min |
| v_sys_max |
| v_dia_max |
| v_sys_over_dia |
| v_sys_dia_spread |
| v_sys_spread |
| v_dia_spread |
| v_sys_stdev |
| v_dia_stdev |
| v_sys_dia_spread_stdev |
| v_12mon |
| n_comorbs |
| comorb_diabetes |
| comorb_chf |
| comorb_copd |
| comorb_mi |
| comorb_esrd |
| comorb_malignant |
| comorb_stroke |
| comorb_mntl_hlth |
| comorb_ltd_life |
| comorb_adv_ev |
| EF |
| MCHC |
| LYMPH% |
| ABS EOSIN |
| RBC |
| MCH |
| ABS MONO |
| MONO% |
| ABS LYMPH |
| BASO% |
| MPV |
| HEMATOCRIT |
| MCV |
| WBC |
| NEUT% |
| EOSIN% |
| RDW-CV |
| PLATELET COUNT |
| HEMOGLOBIN |
| ABS NEUT (ANC) |
| ABS BASO |
| PHOSPHORUS |
| ALBUMIN |
| POTASSIUM |
| GLUCOSE |
| CREATININE |
| EGFR-AFRICAN AMERICAN |
| CHLORIDE |
| EGFR-ALL OTHER RACES |
| SODIUM |
| URIC ACID |
| ANION GAP |
| CALCIUM |
| BUN |
| CO2 |
| VITAMIN D 25 HYDROXY |
| ABSOLUTE NRBC |
| PROTEIN, URINE |
| RBC, URINE |
| PH, URINE |
| WBC, URINE |
| SPECIFIC GRAVITY, UR |
| CREATININE, UR RANDOM (UCRR) |
| PROTEIN, TOTAL |
| BILIRUBIN, TOTAL |
| ALT |
| AST |
| ALKALINE PHOSPHATASE |
| HEMOGLOBIN A1C |
| BILIRUBIN, CONJUG |
| NUCLEATED REDS |
| TIBC |
| IRON |
| FERRITIN |
| VITAMIN B12 |
| NT PRO BNP |
| MAGNESIUM |
| LIPASE |
| PT SEC |
| PT INR |
| APTT |
| UROBILINOGEN |
| LD |
| TSH |
| NON HDL CHOLESTEROL |
| HDL CHOLESTEROL |
| TRIGLYCERIDE |
| LDL CHOLESTEROL |
| CHOLESTEROL, TOTAL |
| LDL HDL RATIO |
| VLDL CHOLESTEROL |
| FASTING TIME |
| TC HDL RATIO |
| FIO2 |
| PRE RESP RATE |
| POST HEART RATE |
| LPM |
| RESPIRATORY RATE (POST) |
| PRE HEART RATE |
| O2 SPO2 |
| TROPONIN T |
| BASE EXCESS, VENOUS |
| BICARBONATE, VENOUS |
| PH, VENOUS |
| PCO2, VENOUS |
| LACTATE |
| TEMPERATURE, BODY, VENOUS |
| ESTIMATED AVERAGE GLUCOSE |
| FREE T4 |
| CRP |
| CK |
| TEMPERATURE, BODY, ARTERIAL |
| PO2, VENOUS |
| ABS. NEUT(ANC) |
| ABS. EOSIN |
| RDW |
| RDW-SD |
| SEG NEUTROPHIL |
| BASOPHIL |
| LYMPHOCYTE |
| ABS. MONO |
| ABS. LYMPH |
| ABS. BASO |
| IMMATURE GRANS # |
| EOSINOPHIL |
| IMMATURE GRANS |
| MONOCYTE |
| EGFR |
| CK MB % |
| HGB |
| ACTIVATED CLOTTING TIME (POCT) |
| ALBUMIN, URINE RANDOM |
| GLUCOSE, POINT OF CARE |
| ALBUMIN/CREAT RATIO |
| PH UA (POCT) |
| SPECIFIC GRAVITY UA (POCT) |
| UROBILINOGEN UA (POCT) |
| PROTEIN UA (POCT) |
| GLUCOSE, URINE |
| PH, ARTERIAL |
| HEMOGLOBIN TOTAL, WHOLE BLOOD |
| SODIUM, WHOLE BLOOD |
| PCO2, ARTERIAL |
| CARBOXYHEMOGLOBIN, ARTERIAL |
| METHEMOGLOBIN, ARTERIAL |
| BICARBONATE, ARTERIAL |
| HEMATOCRIT, WHOLE BLOOD |
| CALCIUM IONIZED, WHOLE BLOOD |
| PO2, ARTERIAL |
| BASE EXCESS, ARTERIAL |
| CO2 CONTENT, ARTERIAL |
| POTASSIUM, WHOLE BLOOD |
| PH, TEMP CORRECTED, ARTERIAL |
| GLUCOSE, WHOLE BLOOD |
| PO2, TEMP CORRECTED, ARTERIAL |
| PCO2, TEMP CORRECTED, ARTERIAL |
| OXYHEMOGLOBIN, ARTERIAL |
| CO2 CONTENT, VENOUS |
| PH, TEMP CORRECTED, VENOUS |
| OXYHEMOGLOBIN, VENOUS |
| PO2, TEMP CORRECTED, VENOUS |
| METHEMOGLOBIN, VENOUS |
| PCO2, TEMP CORRECTED, VENOUS |
| CARBOXYHEMOGLOBIN, VENOUS |
| ANC(INCLUDESEG+BAND) |
| IBW |
| PATIENT HEIGHT |
| TOTAL FREQUENCY (BPM) |
| SP02 |
| INR (POCT) |
| BUN/CREATININE RATIO |
| GLUCOSE METER |
| Deceased_Indicator |
| Hispanic_Indicator |
| Interpreter_Needed_Indicator |
| Appointments_Completed_Number |
| Appointment_No_Show_Number |
| age |
| c_hshlds_tot |
| c_hshlds_tot_lt_10k |
| c_hshlds_tot_10k_to_15k |
| c_hshlds_tot_15k_to_25k |
| c_hshlds_tot_25k_to_35k |
| c_hshlds_tot_35k_to_50k |
| c_hshlds_tot_50k_to_75k |
| c_hshlds_tot_75k_to_100k |
| c_hshlds_tot_100k_to_150k |
| c_hshlds_tot_150k_to_200k |
| c_hshlds_tot_200kplus |
| c_hshlds_Median income |
| c_hshlds_Mean income |
| c_hshlds_pct_alloc_hshld_inc_12 mon |
| c_fam_tot |
| c_fam_tot_lt_10k |
| c_fam_tot_10k_to_15k |
| c_fam_tot_15k_to_25k |
| c_fam_tot_25k_to_35k |
| c_fam_tot_35k_to_50k |
| c_fam_tot_50k_to_75k |
| c_fam_tot_75k_to_100k |
| c_fam_tot_100k_to_150k |
| c_fam_tot_150k_to_200k |
| c_fam_tot_200kplus |
| c_fam_Median income |
| c_fam_Mean income |
| c_fam_pct_alloc_fam_inc_12_mon |
| c_mrrd fam_tot |
| c_mrrd fam_tot_lt_10k |
| c_mrrd fam_tot_10k_to_15k |
| c_mrrd fam_tot_15k_to_25k |
| c_mrrd fam_tot_25k_to_35k |
| c_mrrd fam_tot_35k_to_50k |
| c_mrrd fam_tot_50k_to_75k |
| c_mrrd fam_tot_75k_to_100k |
| c_mrrd fam_tot_100k_to_150k |
| c_mrrd fam_tot_150k_to_200k |
| c_mrrd fam_tot_200kplus |
| c_mrrd fam_Median income |
| c_nonfam hshlds_tot |
| c_nonfam hshlds_tot_lt_10k |
| c_nonfam hshlds_tot_10k_to_15k |
| c_nonfam hshlds_tot_15k_to_25k |
| c_nonfam hshlds_tot_25k_to_35k |
| c_nonfam hshlds_tot_35k_to_50k |
| c_nonfam hshlds_tot_50k_to_75k |
| c_nonfam hshlds_tot_75k_to_100k |
| c_nonfam hshlds_tot_100k_to_150k |
| c_nonfam hshlds_tot_150k_to_200k |
| c_nonfam hshlds_tot_200kplus |
| c_nonfam hshlds_Median income |
| c_nonfam hshlds_Mean income |
| enc_ABSTRACT_ct |
| enc_ABSTRACT_30d_mean |
| enc_ABSTRACT_90d_mean |
| enc_ABSTRACT_180d_mean |
| enc_ABSTRACT_180_over_90 |
| enc_ANTICOAGULATION VISIT_ct |
| enc_ANTICOAGULATION VISIT_30d_mean |
| enc_ANTICOAGULATION VISIT_90d_mean |
| enc_ANTICOAGULATION VISIT_180d_mean |
| enc_ANTICOAGULATION VISIT_180_over_90 |
| enc_APPOINTMENT_ct |
| enc_APPOINTMENT_30d_mean |
| enc_APPOINTMENT_90d_mean |
| enc_APPOINTMENT_180d_mean |
| enc_APPOINTMENT_180_over_90 |
| enc_BILLING ENCOUNTER_ct |
| enc_BILLING ENCOUNTER_30d_mean |
| enc_BILLING ENCOUNTER_90d_mean |
| enc_BILLING ENCOUNTER_180d_mean |
| enc_BILLING ENCOUNTER_180_over_90 |
| enc_DISTANCE HEALTH_ct |
| enc_DISTANCE HEALTH_30d_mean |
| enc_DISTANCE HEALTH_90d_mean |
| enc_DISTANCE HEALTH_180d_mean |
| enc_DISTANCE HEALTH_180_over_90 |
| enc_EDUCATION_ct |
| enc_EDUCATION_30d_mean |
| enc_EDUCATION_90d_mean |
| enc_EDUCATION_180d_mean |
| enc_EDUCATION_180_over_90 |
| enc_EXTERNAL CLINICAL DOCUMENT(S)_ct |
| enc_EXTERNAL CLINICAL DOCUMENT(S)_30d_mean |
| enc_EXTERNAL CLINICAL DOCUMENT(S)_90d_mean |
| enc_EXTERNAL CLINICAL DOCUMENT(S)_180d_mean |
| enc_EXTERNAL CLINICAL DOCUMENT(S)_180_over_90 |
| enc_EXTERNAL CORRESPONDENCE_ct |
| enc_EXTERNAL CORRESPONDENCE_30d_mean |
| enc_EXTERNAL CORRESPONDENCE_90d_mean |
| enc_EXTERNAL CORRESPONDENCE_180d_mean |
| enc_EXTERNAL CORRESPONDENCE_180_over_90 |
| enc_HEALTH MAINTENANCE LETTER_ct |
| enc_HEALTH MAINTENANCE LETTER_30d_mean |
| enc_HEALTH MAINTENANCE LETTER_90d_mean |
| enc_HEALTH MAINTENANCE LETTER_180d_mean |
| enc_HEALTH MAINTENANCE LETTER_180_over_90 |
| enc_HEMONC ORDERS ONLY_ct |
| enc_HEMONC ORDERS ONLY_30d_mean |
| enc_HEMONC ORDERS ONLY_90d_mean |
| enc_HEMONC ORDERS ONLY_180d_mean |
| enc_HEMONC ORDERS ONLY_180_over_90 |
| enc_HISTORY_ct |
| enc_HISTORY_30d_mean |
| enc_HISTORY_90d_mean |
| enc_HISTORY_180d_mean |
| enc_HISTORY_180_over_90 |
| enc_HOME CARE UPDATE_ct |
| enc_HOME CARE UPDATE_30d_mean |
| enc_HOME CARE UPDATE_90d_mean |
| enc_HOME CARE UPDATE_180d_mean |
| enc_HOME CARE UPDATE_180_over_90 |
| enc_HOME CARE VISIT_ct |
| enc_HOME CARE VISIT_30d_mean |
| enc_HOME CARE VISIT_90d_mean |
| enc_HOME CARE VISIT_180d_mean |
| enc_HOME CARE VISIT_180_over_90 |
| enc_HOSPITAL ENCOUNTER_ct |
| enc_HOSPITAL ENCOUNTER_30d_mean |
| enc_HOSPITAL ENCOUNTER_90d_mean |
| enc_HOSPITAL ENCOUNTER_180d_mean |
| enc_HOSPITAL ENCOUNTER_180_over_90 |
| enc_INFUSION CENTER_ct |
| enc_INFUSION CENTER_30d_mean |
| enc_INFUSION CENTER_90d_mean |
| enc_INFUSION CENTER_180d_mean |
| enc_INFUSION CENTER_180_over_90 |
| enc_LETTER (OUT)_ct |
| enc_LETTER (OUT)_30d_mean |
| enc_LETTER (OUT)_90d_mean |
| enc_LETTER (OUT)_180d_mean |
| enc_LETTER (OUT)_180_over_90 |
| enc_LETTERS (IN)_ct |
| enc_LETTERS (IN)_30d_mean |
| enc_LETTERS (IN)_90d_mean |
| enc_LETTERS (IN)_180d_mean |
| enc_LETTERS (IN)_180_over_90 |
| enc_MC GET MEDICAL ADVICE_ct |
| enc_MC GET MEDICAL ADVICE_30d_mean |
| enc_MC GET MEDICAL ADVICE_90d_mean |
| enc_MC GET MEDICAL ADVICE_180d_mean |
| enc_MC GET MEDICAL ADVICE_180_over_90 |
| enc_MC PATIENT MSG_ct |
| enc_MC PATIENT MSG_30d_mean |
| enc_MC PATIENT MSG_90d_mean |
| enc_MC PATIENT MSG_180d_mean |
| enc_MC PATIENT MSG_180_over_90 |
| enc_NURSE TRIAGE_ct |
| enc_NURSE TRIAGE_30d_mean |
| enc_NURSE TRIAGE_90d_mean |
| enc_NURSE TRIAGE_180d_mean |
| enc_NURSE TRIAGE_180_over_90 |
| enc_NURSE VISIT_ct |
| enc_NURSE VISIT_30d_mean |
| enc_NURSE VISIT_90d_mean |
| enc_NURSE VISIT_180d_mean |
| enc_NURSE VISIT_180_over_90 |
| enc_OFFICE VISIT_ct |
| enc_OFFICE VISIT_30d_mean |
| enc_OFFICE VISIT_90d_mean |
| enc_OFFICE VISIT_180d_mean |
| enc_OFFICE VISIT_180_over_90 |
| enc_OFFICE VISIT AC_ct |
| enc_OFFICE VISIT AC_30d_mean |
| enc_OFFICE VISIT AC_90d_mean |
| enc_OFFICE VISIT AC_180d_mean |
| enc_OFFICE VISIT AC_180_over_90 |
| enc_OFFICE VISIT CP_ct |
| enc_OFFICE VISIT CP_30d_mean |
| enc_OFFICE VISIT CP_90d_mean |
| enc_OFFICE VISIT CP_180d_mean |
| enc_OFFICE VISIT CP_180_over_90 |
| enc_OFFICE VISIT FLA_ct |
| enc_OFFICE VISIT FLA_30d_mean |
| enc_OFFICE VISIT FLA_90d_mean |
| enc_OFFICE VISIT FLA_180d_mean |
| enc_OFFICE VISIT FLA_180_over_90 |
| enc_OFFICE VISIT OPHT_ct |
| enc_OFFICE VISIT OPHT_30d_mean |
| enc_OFFICE VISIT OPHT_90d_mean |
| enc_OFFICE VISIT OPHT_180d_mean |
| enc_OFFICE VISIT OPHT_180_over_90 |
| enc_ORDERS ONLY_ct |
| enc_ORDERS ONLY_30d_mean |
| enc_ORDERS ONLY_90d_mean |
| enc_ORDERS ONLY_180d_mean |
| enc_ORDERS ONLY_180_over_90 |
| enc_ORDERS ONLY FLA_ct |
| enc_ORDERS ONLY FLA_30d_mean |
| enc_ORDERS ONLY FLA_90d_mean |
| enc_ORDERS ONLY FLA_180d_mean |
| enc_ORDERS ONLY FLA_180_over_90 |
| enc_OT/PT/SPEECH VISIT_ct |
| enc_OT/PT/SPEECH VISIT_30d_mean |
| enc_OT/PT/SPEECH VISIT_90d_mean |
| enc_OT/PT/SPEECH VISIT_180d_mean |
| enc_OT/PT/SPEECH VISIT_180_over_90 |
| enc_PAT_ct |
| enc_PAT_30d_mean |
| enc_PAT_90d_mean |
| enc_PAT_180d_mean |
| enc_PAT_180_over_90 |
| enc_PATIENT OUTREACH_ct |
| enc_PATIENT OUTREACH_30d_mean |
| enc_PATIENT OUTREACH_90d_mean |
| enc_PATIENT OUTREACH_180d_mean |
| enc_PATIENT OUTREACH_180_over_90 |
| enc_PATIENT UPDATE_ct |
| enc_PATIENT UPDATE_30d_mean |
| enc_PATIENT UPDATE_90d_mean |
| enc_PATIENT UPDATE_180d_mean |
| enc_PATIENT UPDATE_180_over_90 |
| enc_PHARMACY VISIT_ct |
| enc_PHARMACY VISIT_30d_mean |
| enc_PHARMACY VISIT_90d_mean |
| enc_PHARMACY VISIT_180d_mean |
| enc_PHARMACY VISIT_180_over_90 |
| enc_PROCEDURE_ct |
| enc_PROCEDURE_30d_mean |
| enc_PROCEDURE_90d_mean |
| enc_PROCEDURE_180d_mean |
| enc_PROCEDURE_180_over_90 |
| enc_PROCEDURE PASS_ct |
| enc_PROCEDURE PASS_30d_mean |
| enc_PROCEDURE PASS_90d_mean |
| enc_PROCEDURE PASS_180d_mean |
| enc_PROCEDURE PASS_180_over_90 |
| enc_RADIOLOGY_ct |
| enc_RADIOLOGY_30d_mean |
| enc_RADIOLOGY_90d_mean |
| enc_RADIOLOGY_180d_mean |
| enc_RADIOLOGY_180_over_90 |
| enc_RECONCILED OUTSIDE DATA_ct |
| enc_RECONCILED OUTSIDE DATA_30d_mean |
| enc_RECONCILED OUTSIDE DATA_90d_mean |
| enc_RECONCILED OUTSIDE DATA_180d_mean |
| enc_RECONCILED OUTSIDE DATA_180_over_90 |
| enc_REFILL_ct |
| enc_REFILL_30d_mean |
| enc_REFILL_90d_mean |
| enc_REFILL_180d_mean |
| enc_REFILL_180_over_90 |
| enc_REFILL - MYCHART_ct |
| enc_REFILL - MYCHART_30d_mean |
| enc_REFILL - MYCHART_90d_mean |
| enc_REFILL - MYCHART_180d_mean |
| enc_REFILL - MYCHART_180_over_90 |
| enc_REFILL CP_ct |
| enc_REFILL CP_30d_mean |
| enc_REFILL CP_90d_mean |
| enc_REFILL CP_180d_mean |
| enc_REFILL CP_180_over_90 |
| enc_REFILL FLA_ct |
| enc_REFILL FLA_30d_mean |
| enc_REFILL FLA_90d_mean |
| enc_REFILL FLA_180d_mean |
| enc_REFILL FLA_180_over_90 |
| enc_RESULTS ONLY_ct |
| enc_RESULTS ONLY_30d_mean |
| enc_RESULTS ONLY_90d_mean |
| enc_RESULTS ONLY_180d_mean |
| enc_RESULTS ONLY_180_over_90 |
| enc_RESULTS ONLY FLA_ct |
| enc_RESULTS ONLY FLA_30d_mean |
| enc_RESULTS ONLY FLA_90d_mean |
| enc_RESULTS ONLY FLA_180d_mean |
| enc_RESULTS ONLY FLA_180_over_90 |
| enc_SOCIAL WORK_ct |
| enc_SOCIAL WORK_30d_mean |
| enc_SOCIAL WORK_90d_mean |
| enc_SOCIAL WORK_180d_mean |
| enc_SOCIAL WORK_180_over_90 |
| enc_SURGERY_ct |
| enc_SURGERY_30d_mean |
| enc_SURGERY_90d_mean |
| enc_SURGERY_180d_mean |
| enc_SURGERY_180_over_90 |
| enc_TELEPHONE_ct |
| enc_TELEPHONE_30d_mean |
| enc_TELEPHONE_90d_mean |
| enc_TELEPHONE_180d_mean |
| enc_TELEPHONE_180_over_90 |
| enc_TELEPHONE CP_ct |
| enc_TELEPHONE CP_30d_mean |
| enc_TELEPHONE CP_90d_mean |
| enc_TELEPHONE CP_180d_mean |
| enc_TELEPHONE CP_180_over_90 |
| enc_TELEPHONE FLA_ct |
| enc_TELEPHONE FLA_30d_mean |
| enc_TELEPHONE FLA_90d_mean |
| enc_TELEPHONE FLA_180d_mean |
| enc_TELEPHONE FLA_180_over_90 |
| enc_TRAVEL_ct |
| enc_TRAVEL_30d_mean |
| enc_TRAVEL_90d_mean |
| enc_TRAVEL_180d_mean |
| enc_TRAVEL_180_over_90 |
| enc_VISIT (SP) OFFICE_ct |
| enc_VISIT (SP) OFFICE_30d_mean |
| enc_VISIT (SP) OFFICE_90d_mean |
| enc_VISIT (SP) OFFICE_180d_mean |
| enc_VISIT (SP) OFFICE_180_over_90 |
| cntry__ALBANIA |
| cntry__ANTIGUA |
| cntry__ARGENTINA |
| cntry__ARUBA - AW |
| cntry__AUSTRALIA |
| cntry__AUSTRIA |
| cntry__AZERBAIJAN |
| cntry__BAHAMAS |
| cntry__BAHRAIN |
| cntry__BANGLADESH |
| cntry__BARBADOS |
| cntry__BELGIUM |
| cntry__BELIZE |
| cntry__BERMUDA |
| cntry__BOLIVIA |
| cntry__BRAZIL |
| cntry__BRITISH VIRGIN ISLANDS |
| cntry__CAMBODIA |
| cntry__CANADA |
| cntry__CAYMAN ISLANDS |
| cntry__CHILE |
| cntry__CHINA PEOPLES REPUBLIC |
| cntry__COLOMBIA |
| cntry__COSTA RICA |
| cntry__CROATIA |
| cntry__CURACAO |
| cntry__DOMINICAN REPUBLIC |
| cntry__ECUADOR |
| cntry__EGYPT |
| cntry__EL SALVADOR |
| cntry__FINLAND |
| cntry__FRANCE |
| cntry__GERMANY |
| cntry__GREECE |
| cntry__GUATEMALA |
| cntry__GUYANA |
| cntry__HAITI |
| cntry__HONDURAS |
| cntry__HONG KONG |
| cntry__HUNGARY |
| cntry__INDIA |
| cntry__INDONESIA |
| cntry__IRAN |
| cntry__ISRAEL |
| cntry__ITALY |
| cntry__JAMAICA |
| cntry__JAPAN |
| cntry__JORDAN |
| cntry__KENYA |
| cntry__KUWAIT |
| cntry__LEBANON |
| cntry__MEXICO |
| cntry__MONACO |
| cntry__NAMIBIA |
| cntry__NEPAL |
| cntry__NETHERLANDS |
| cntry__NEVIS |
| cntry__NEW ZEALAND |
| cntry__NICARAGUA |
| cntry__NIGERIA |
| cntry__NORWAY |
| cntry__PAKISTAN |
| cntry__PALESTINE |
| cntry__PANAMA |
| cntry__PARAGUAY |
| cntry__PERU |
| cntry__PHILIPPINES |
| cntry__QATAR |
| cntry__RUSSIA |
| cntry__SAINT KITTS |
| cntry__SAINT LUCIA |
| cntry__SAUDI ARABIA |
| cntry__SINGAPORE |
| cntry__SOUTH KOREA |
| cntry__SPAIN |
| cntry__SWITZERLAND |
| cntry__THAILAND |
| cntry__TONGA |
| cntry__TRINIDAD AND TOBAGO |
| cntry__TURKEY |
| cntry__TURKMENISTAN |
| cntry__TURKS AND CAICOS ISLANDS |
| cntry__TUVALU |
| cntry__UGANDA |
| cntry__UKRAINE |
| cntry__UNITED ARAB EMIRATES |
| cntry__UNITED KINGDOM |
| cntry__UNITED STATES OF AMERICA |
| cntry__URUGUAY |
| cntry__UZBEKISTAN |
| cntry__VANUATU |
| cntry__VATICAN CITY |
| cntry__VENEZUELA |
| cntry__WESTERN SAHARA |
| empl_stat__FULL TIME |
| empl_stat__NOT EMPLOYED |
| empl_stat__ON ACTIVE MILITARY DUTY |
| empl_stat__PART TIME |
| empl_stat__RETIRED |
| empl_stat__SELF EMPLOYED |
| empl_stat__STUDENT - FULL TIME |
| empl_stat__STUDENT - PART TIME |
| empl_stat__UNKNOWN |
| eth_grp__DECLINED |
| eth_grp__HISPANIC |
| eth_grp__NOT HISPANIC |
| eth_grp__UNAVAILABLE |
| gend__FEMALE |
| gend__MALE |
| gend__UNKNOWN |
| gend__X |
| lang__AFRIKAANS |
| lang__AKAN |
| lang__ALBANIAN |
| lang__AMHARIC |
| lang__ARABIC |
| lang__ARMENIAN |
| lang__AZERBAIJANI |
| lang__BENGALI |
| lang__BOSNIAN |
| lang__BULGARIAN |
| lang__BURMESE |
| lang__CAMBODIAN |
| lang__CANTONESE |
| lang__CHINESE |
| lang__CREOLE |
| lang__CROATIAN |
| lang__CZECH |
| lang__DARI PERSIAN |
| lang__DUTCH |
| lang__ENGLISH |
| lang__ESTONIAN |
| lang__ETHIOPIAN |
| lang__FARSI |
| lang__FIJIAN |
| lang__FINNISH |
| lang__FRENCH |
| lang__FRENCH CANADIAN |
| lang__FRENCH CREOLE |
| lang__GERMAN |
| lang__GREEK |
| lang__GUJARATHI |
| lang__HAITIAN CREOLE |
| lang__HEBREW |
| lang__HINDI |
| lang__HINDU |
| lang__HMONG |
| lang__HUNGARIAN |
| lang__IBO |
| lang__ICELANDIC |
| lang__INDIAN |
| lang__INDONESIAN |
| lang__IRANIAN |
| lang__ITALIAN |
| lang__JAPANESE |
| lang__KANNADA |
| lang__KHMER |
| lang__KINYARWAND |
| lang__KOREAN |
| lang__KRU |
| lang__KURDISH |
| lang__LAO |
| lang__LATVIAN |
| lang__LITHUANIAN |
| lang__LOAOTIAN |
| lang__MACEDONIAR |
| lang__MALAYALAM |
| lang__MANDARIN |
| lang__MARATHI |
| lang__NAJDI |
| lang__NEPALI |
| lang__NIGERIAN PIDGIN ENGLISH |
| lang__NORWEGIAN |
| lang__OROMO |
| lang__OTHER |
| lang__PAKISTANI |
| lang__PANGASINAN |
| lang__PANJABI |
| lang__PERSIAN |
| lang__POLISH |
| lang__PORTUGESE |
| lang__PUSHTO |
| lang__ROMANIAN |
| lang__RUSSIAN |
| lang__SERBCROATIAN |
| lang__SERBIA |
| lang__SERBIAN |
| lang__SICILIAN |
| lang__SIGN LANGUAGE |
| lang__SINHALA |
| lang__SLOVAK |
| lang__SLOVENE |
| lang__SOMALIA |
| lang__SPANISH |
| lang__SWAHILI |
| lang__SYRIAC |
| lang__TAGALOG-FILIPINO |
| lang__TAMIL |
| lang__THAI |
| lang__TURKISH |
| lang__UKRANIAN |
| lang__UNKNOWN |
| lang__URDU |
| lang__VIETNAMESE |
| lang__YIDDISH |
| lang__YORUBA |
| lang__YUGOSLAVIAN |
| mrtl_stat__DIVORCED |
| mrtl_stat__DOMESTIC PARTNER |
| mrtl_stat__LEGALLY SEPARATED |
| mrtl_stat__MARRIED |
| mrtl_stat__OTHER |
| mrtl_stat__PATIENT REFUSED |
| mrtl_stat__SINGLE |
| mrtl_stat__UNKNOWN |
| mrtl_stat__WIDOWED |
| race__AMERICAN INDIAN/ALASKA NATIVE |
| race__ASIAN |
| race__BLACK |
| race__DECLINED |
| race__HISPANIC/LATINO |
| race__MULTIRACIAL/MULTICULTURAL |
| race__OTHER |
| race__UNAVAILABLE |
| race__WHITE |
| class_ACE inhibitors |
| class_Alpha blockers |
| class_Alpha-2 Receptor Agonists |
| class_Angiotensin II receptor blockers |
| class_Beta-blockers |
| class_Blood vessel dilators (vasodilators) |
| class_Calcium channel blockers |
| class_Combined alpha and beta-blockers |
| class_Loop diuretic |
| class_Potassium-sparing diuretics |
| class_Thiazide diuretics |
| generic_lisinopril |
| generic_furosemide |
| generic_metoprolol tartrate |
| generic_hydrochlorothiazide |
| generic_metoprolol succinate |
| generic_carvedilol |
| generic_spironolactone |
| generic_atenolol |
| generic_valsartan |
| generic_triamterene |
| generic_nifedipine |
| generic_chlorthalidone |
| generic_ramipril |
| generic_olmesartan |
| generic_enalapril maleate |
| generic_timolol maleate |
| generic_metolazone |
| generic_bumetanide |
| generic_captopril |
| generic_nicardipine |
| generic_nadolol |
| generic_telmisartan |
| generic_felodipine |
| generic_candesartan |
| generic_eplerenone |
| generic_indapamide |
